# Supplementary material for: The interactome of intact mitochondria by cross-linking mass spectrometry provides evidence for coexisting respiratory supercomplexes
Source: Mol Cell Proteomics. 2017 Dec 8;17(2):216–32. doi: 10.1074/mcp.RA117.000470 (PMC5795388; doi:10.1074/mcp.RA117.000470)
Supplement: Supplemental Data [file supp_RA117.000470_133922_0_supp_23978_fzffwf.zip › spectra_annotation/mito_DR_spectra_annotation/287-1-2-1-3-1.pdf]

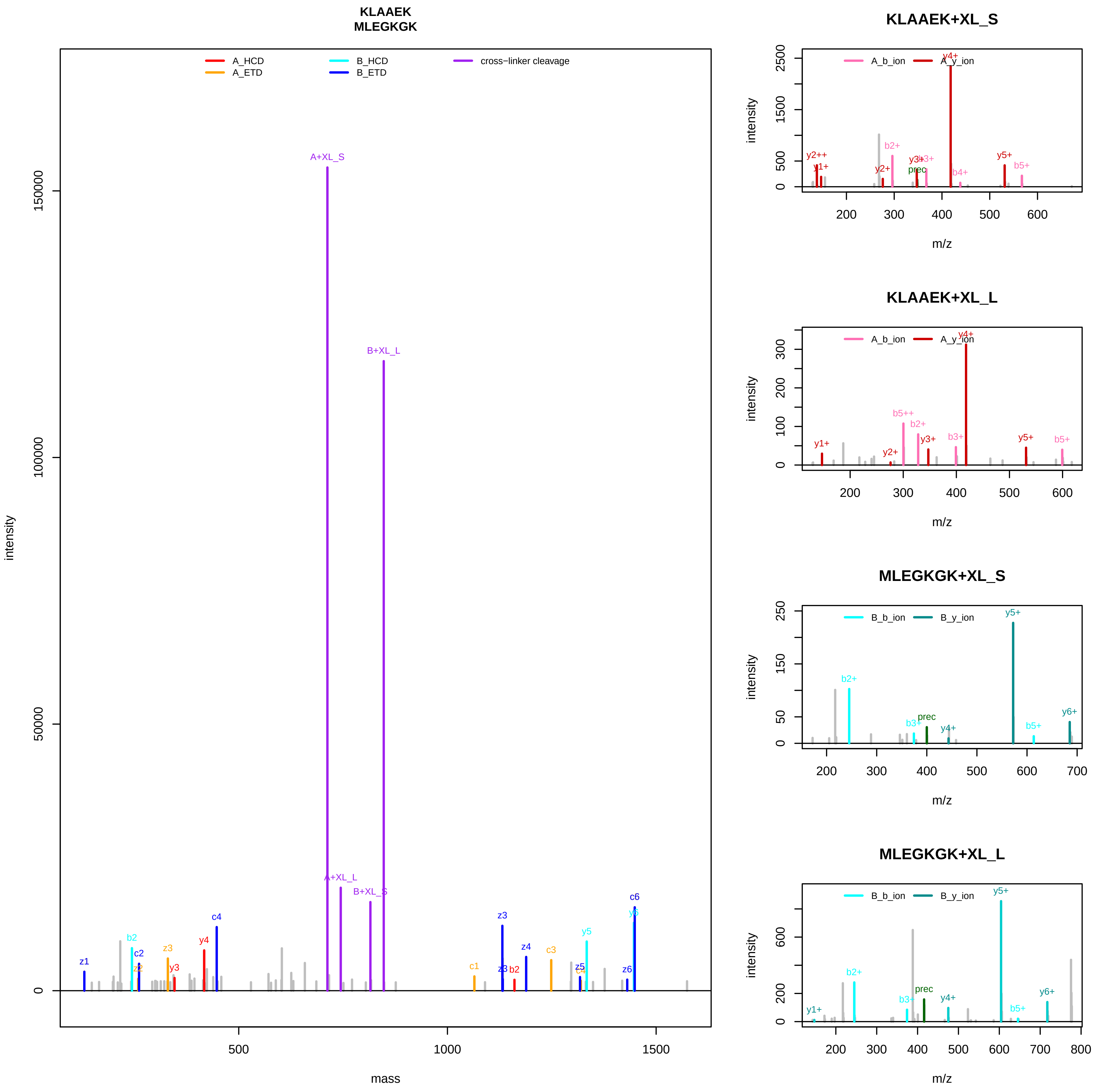
KLAAEK
MLEGKGK
A_HCD
A_ETD
B_HCD
B_ETD
cross−linker cleavage
A+XL_S
B+XL_L
A+XL_L
B+XL_S
z1
b2
c2
z2
z3
y3
y4
c4
c1
z3
b2
z4
c3
z5
c4
y5
z6
y6
c6
0
50000
100000
150000
500
1000
1500
intensity
mass
KLAAEK+XL_S
A_b_ion
A_y_ion
KLAAEK+XL_L
MLEGKGK+XL_S
B_b_ion
B_y_ion
MLEGKGK+XL_L
prec
m/z
intensity
